# A comparative analysis on human resources among the specialized ophthalmic medical institutions in China

**DOI:** 10.1186/s12960-020-00471-1

**Published:** 2020-04-16

**Authors:** Leilei Zhan, Neha Safaya, Hana Erkou, Lei An, Zhifeng Wang, Jingjing Feng, Xiao Xu

**Affiliations:** 1 WHO Collaborating Centre for the Prevention of Blindness, Department of Nursing Administration and Rehabilitation Research, National Institute of Hospital Administration, National Health Commission, Building 3, Room 502, No.6, Shoutinan Road, Haidian District, Beijing, 100044 People’s Republic of China; 2 Heller School for Social Policy and Management, Brandeis University, Waltham, United States of America; 3 School of Public Health, Peking University Health Science Center, Beijing, People’s Republic of China

**Keywords:** Human resource, Ophthalmology, Medical institution, China

## Abstract

**Background:**

This study compares perspectives on specialized ophthalmic medical institutions, identifies the gaps in property and geographic offerings, and explores the ways that ophthalmic medical institutions can better allocate resources. The results of this research will increase patient’s access to equitable and high-quality ophthalmic care in China.

**Methods:**

The data for this research was gathered from the *Survey of China National Eye Care Capacity and Resource* for the year 2015. The paper specified the number, professional level of expertise, and educational background of ophthalmic health personnel. The authors of the paper analyzed and compared the differences in ophthalmic care in public vs. private and urban vs. rural regions in China. Descriptive statistics were used.

**Results:**

Of the 395 specialized ophthalmic hospitals surveyed, 332 were private medical institutions (84%), and 63 were public (16%). Of the 26 607 ophthalmic personnel surveyed, working in specialized ophthalmic hospitals, 17 561 were in private hospitals (66%) and 9 046 were in public ones (34%). Furthermore, 22 578 of those personnel worked in urban ophthalmic institutions (85%) and 4 029 worked in rural ones (15%). As for regional differences, 14 090 personnel were located in eastern China (53%), 8 828 in central regions (33%), and 3 689 in the western regions (14%).

**Conclusions:**

Public ophthalmic medical institutions still face challenges in providing equitable and widespread care. The availability of well-staffed health centers varies significantly by region. These variations impact resource allocation and directly lead to inequalities and inaccessibility of health services in certain regions of China.

## Introduction

Ophthalmic health personnel mostly include ophthalmologists, ophthalmic assistants, scientific researchers, management personnel, nursing staff, optometrists, opticians, and technicians. The diversity in and depth of human resource staffing is an important indicator for measuring a facility’s ability to provide comprehensive services and its capacity to treat patients. The personnel must have been trained on how to teach blindness prevention methods and treat blindness among their regional population. Therefore, the Chinese government, at various levels, has made great efforts to build a strong ophthalmic human resource system. Although great strides have been made, issues related to adequate human resources staffing still exist. In 2016, the population of rural and urban areas in China were 583 million rural (42.65%) and 793 million urban (57.35%), and those of eastern, central, and western regions were 573 million (41.46%), 432 million (31.27%), and 377 million (27.27%) [1]. The allocation of human resources for medical institutions greatly favored urban regions and still had difficulty meeting the needs of larger populations.

Efforts to increase ophthalmic disease and blindness prevention measures in China have witnessed notable developments under the leadership of the Chinese government. The qualifications needed to become an ophthalmic professional have been raised to meet the demands of the population. For example, formerly, most physicians in rural hospitals were only required to have a technical secondary school education or below, but currently, it has been increased to a bachelor’s degree. Previously, the terminal degree for nursing was a technical secondary school, which has been upgraded to include everything up to doctoral degree in Nursing. Although there is not a universal degree requirement to qualify for conducting scientific research and managing personnel in China, the average level of education has been rising from technical degree to bachelor’s degree. With this change, ophthalmic service capacity in medical institutions, especially at the grassroots level, has been increasing significantly. As a result of healthcare reform in China in recent years, public general hospitals have been encountering many challenges. From 2000 to 2014, the national government issued dozens of official documents to regulate the establishment and management of medical institutions. It advocated for the introduction of social capital to invest in medical service businesses and required public and non-public medical institutions to regulate and distribute responsibilities.

Although there are many papers studying human resource allocation in Chinese medical institutions, few have focused on specialized ophthalmic medical institutions or made comparative studies [2–6]. Therefore, by focusing on ophthalmic medical institutions, this paper aims to assess the current state of ophthalmic human resources in China and to identify the gaps in resource provision between public and private institutions and the differences in care between urban and rural regions, and across regions. The purpose of this paper is to provide a basis for healthcare reform in China as it relates to ophthalmic care and to optimize ophthalmic health resource allocation in China.

## Methods

### Data source

Led by the former Chinese National Health and Family Planning Commission, the *Survey of China National Eye Care Capacity and Resource* was carried out across China in 2015. Each hospital starting from the county level and above registered in China before 31 December 2014, as required by the national government, reported all data related to ophthalmic health resources online for that year, including 31 provinces (including autonomous regions and municipalities) and the Xinjiang Production and Construction Corps. This survey was a standardized questionnaire to be completed online and supervised by provincial health and family planning commission in each province. Special personnel were designated to fill in questionnaires, and the head of the ophthalmology department took charge of submitting the finalized data. The survey period was from 1 January 2014 to 31 December 2014. Among the 6341 medical institutions effectively enrolled into this study, 395 were specialized ophthalmic hospitals (6.2%). Country-level data on human resources in specialized ophthalmic medical institutions were used for this study. The 2010 and 2012 data was obtained from *China’s Health Statistics Yearbook 2016* [7]*.*

### Main indicators

Health personnel working in specialized ophthalmic institutions were divided into seven categories, including physicians, physician assistants, scientific research and management personnel, nursing staff, optometrists, opticians, and technicians. This paper focused on three main human resource categories to make comparison and analysis: physicians, scientific research and management personnel, and nursing staff. Three indicators associated with these categories were identified in this study, including the number of staff, academic degree, and professional rank.

All medical institutions studied were divided into three categories, including public and private, urban and rural, and geographic region, with the inclusion criteria as follows:

1. Public medical institution refers to institutions that are registered as state-owned or collective-owned. Private medical institution refers to institutions registered as anything other than public, including joint operation, joint stock partnership, private-owned hospitals, or hospitals invested in by citizens from Taiwan, Hong Kong, Macao, or foreign countries [7]. Since medical institutions were divided into three categories in line with the Medical Institution Practice License, non-profit (public), non-profit (private), and profit-making, non-profit (private), profit-making medical institutions were combined to be viewed as private medical institutions.
2. Urban medical institutions are referring to institutions located in cities (including districts). Rural institutions are institutions located in counties (including county-level cities), including township health centers and village clinics [7].
3. Geographic locations of medical institutions surveyed were divided into three regions: eastern (including Beijing, Tianjin, Hebei, Liaoning, Shanghai, Jiangsu, Zhejiang, Fujian, Shandong, Guangdong, and Hainan province), central (including Shanxi, Jilin, Heilong Jiang, Anhui, Jiangxi, Henan, Hubei, and Hunan), and western (including Inner Mongolia, Chongqing, Guangxi, Sichuan, Guizhou, Yunnan, Tibet, Shaanxi, Gansu, Qinghai, Ningxia, and Xinjiang Uygur Autonomous Region) [7].

### Statistical method

Data cleaning and descriptive analysis were carried out using the SPSS19.0 statistical software. A consistency check was conducted by using singular values and illogical data, screening data variables, and then conducting a data review by phone calls to resolve data queries and ensure data quality.

## Results

### The developing trend of specialized ophthalmic medical institutions in China

Table 1 shows the rapid development, in terms of numbers, of specialized ophthalmic medical institutions beginning in 2010 through 2014. The number of specialized ophthalmic medical institutions increased by 50%, health personnel increased by 31%, and outpatient service and emergency treatment increased by 158%. Though the percentage change was great, the total quantifiable growth of medical institutions was not as significant.

**Table 1:** Specialized ophthalmic medical institutions from 2010 to 2014

Year	Institutions	Health personnel	Outpatient service and emergency treatment
2010	263 (0.03%)	20 311 (0.25%)	10 887 249 (0.55%)
2012	326 (0.03%)	26 401 (0.29%)	14 209 454 (0.57%)
2014	395 (0.04%)	26 607 (0.26%)	28 118 102 (0.58%)

Table 2 presents the number and percentage of ophthalmic medical institutions and health personnel in 2014 from three aspects. Of the 395 specialized ophthalmic medical institutions in China, 63 were public (16%) and the remaining 332 were private institutions (84%); 334 were located in urban regions (85%) and the other 61 were in rural areas (15%); and 169 were located in eastern China (43%), 153 were in central China (39%), and the remaining 73 were in the western region (18%).

**Table 2:** Human resource allocation in specialized ophthalmic medical institutions in 2014

Category	Ophthalmic medical institutions	Health personnel	① Physicians	② Physician assistants	③ Scientific research and management fellow	④ Nursing staff	⑤ Optometrists	⑥ Opticians	⑦ Technicians
Type									
Public	63 (16%)	9 046 (34%)	2 595 (29%)	92 (1%)	1 362 (15%)	3 734 (41%)	452 (5%)	315 (3%)	496 (5%)
Private	332 (84%)	17 561 (66%)	3 951 (22%)	556 (3%)	1 622 (9%)	8 010 (46%)	1 182 (7%)	911 (5%)	1 329 (8%)
Administration division									
Urban	334 (85%)	22 578 (85%)	5 659 (25%)	442 (2%)	2 503 (11%)	10 132 45%)	1 387 (6%)	1 035 (5%)	1 420 (6%)
Rural	61 (15%)	4 029 (15%)	887 (22%)	206 (5%)	481 (12%)	1 612 (40%)	247 (6%)	191 (5%)	405 (10%)
Region									
Eastern	169 (43%)	14 090 (53%)	3 537 (25%)	240 (2%)	1 904 (14%)	5 979 (42%)	838 (6%)	620 (4%)	972 (7%)
Central	153 (39%)	8 828 (33%)	2 159 (24%)	281 (3%)	744 (8%)	4 118 (47%)	554 (6%)	415 (5%)	557 (6%)
Western	73 (18%)	3 689 (14%)	850 (23%)	127 (3%)	336 (9%)	1 647 (45%)	242 (7%)	191 (5%)	296 (8%)

Among the 26 607 health personnel working in specialized ophthalmic medical institutions, 66% worked in private institutions, which was as twice as many people working in public ophthalmic medical institutions. Among those 17 561 people in private institutions, nearly one half of them were nursing staff, physicians accounted for 22%, and scientific research and management fellow accounted for 9%, while 9046 people working in public institutions, physicians and scientific research and management fellows, accounted for a larger proportion of human resources, 29% and 15%, respectively.

When comparing urban and rural ophthalmic medical institutions, it is revealed that 85% of health personnel worked in urban regions. Among those personnel, 45% were nursing staff, 25% were physicians, and 11% were scientific research and management fellows. In rural ophthalmic medical institutions, physician assistant and technicians occupy higher proportion.

Based on *China’s Health Statistics Yearbook 2016*, of the 981 432 medical institutions in China in 2014, 36% were located in the eastern region, 32% were in the central region, and the last 32% were in the western region [7]. Among 10 234 213 health personnel in 2014, the percentages of those in the eastern, central, and western regions were 43%, 30%, and 27%, individually. Regarding ophthalmic health personnel, more than half worked in the eastern part, accounting for 53%; 33% worked in the central region; and the remaining 14% worked in the western China.

### Human resource in public and private ophthalmic medical institutions

Table 3 presents the human resource allocation in ophthalmic medical institutions by registration types. In China, qualifications required to become a physician or nursing staff are explicitly stipulated by *Law on Practicing Doctors of the People’s Republic of China*, *Regulations for the Management of Rural Doctors*, *Nurse Management Act of the People’s Republic of China*, and *Measures for the Registration of Practicing Nurses* [8–11]. Doctors and nurses at each level should meet the government’s requirements, pass a qualification examination, and obtain certificates prior to engaging a medical practice. For example, attending doctors must fulfill one of the following requirements: (1) bachelor’s degree or above in medical specialty and 1 year of probation period under the guidance of practicing physicians; (2) having the certificate of physician assistant, college degree in medical specialty, and 2 years’ working in hospitals; or (3) technical secondary school education degree in medical specialty and 5 years’ working in hospitals [8]. And *Regulations for the Management of Rural Doctors* outlines the requirements for rural doctors who have not gained the qualifications or education of urban doctors but are registered in rural medical institutions and allowed to carry out disease prevention and primary healthcare activities. The requirements for rural doctors include the following: (1) technical secondary school education, (2) 20 years’ working in medical institutions, and (3) having certificates of medical training [9].

**Table 3:** Human resource in public and private ophthalmic medical institutions

Category	Physicians		Nursing staff		Scientific research and management fellow	
	Public	Private	Public	Private	Public	Private
Academic degrees						
Doctor’s degree	454 (17%)	120 (3%)	195 (5%)	39 (1%)	141 (10%)	19 (1%)
Master’s degree	941 (36%)	542 (14%)			205 (15%)	45 (3%)
Bachelor’s degree	1 001 (39%)	2 204 (55%)	1 462 (39%)	1 025 (12%)	529 (39%)	574 (35%)
College degree	166 (6%)	877 (22%)	1 739 (46%)	4 378 (54%)	283 (21%)	640 (40%)
Technical secondary school education	28 (1%)	190 (5%)	325 (9%)	2 551 (32%)	121 (9%)	214 (13%)
Others	5 (1%)	18 (1%)	13 (1%)	17 (1%)	83 (6%)	130 (8%)
Professional ranks						
Senior level	934 (36%)	1 190 (30%)	144 (4%)	125 (2%)	127 (9%)	140 (9%)
Medium level	765 (29%)	1 206 (31%)	2 203 (59%)	2 619 (33%)	258 (19%)	286 (18%)
Junior level	896 (35%)	1 555 (39%)	1 258 (34%)	4 982 (62%)	348 (26%)	292 (18%)
Others	**/**	**/**	129 (3%)	284 (3%)	629 (46%)	904 (55%)

In terms of academic degrees, most physicians, 39%, in public institutions had bachelor’s degrees or master’s degrees, 36%, respectively. Alternatively, 55% of physicians working in private institutions had obtained bachelor’s degrees. The educational level of the nursing staff was lower than that of physicians on average. Most nurses working in public institutions had college or bachelor’s degrees, accounting for 46% and 39%, respectively, while 54% of nurses in private institutions had college degrees. Regarding the scientific research and management fellows, most of those working in public institutions had bachelor’s degrees, accounting for 39%, but in private institutions, 40% had college degrees. The proportion of nurses with a higher level of academic training was greater among those working in private institutions.

In terms of professional ranks, the advantageous nature of working for public institutions was not very clear to physicians and scientific research and management fellows. In public institutions, 59% of nursing staff held midlevel positions, while in private ones, 62% of nursing staff held junior-level positions.

### Human resource in urban and rural ophthalmic medical institutions

Table 4 shows the human resources levels divided by administration. Comparing health personnel in urban areas to those in rural areas, half of physicians working in urban regions had bachelor’s degrees. Considering how many physicians had medical degrees, the proportion was 10% in urban institutions and just 1% in rural institutions.

**Table 4:** Human resource in urban and rural ophthalmic medical institutions

Category	Physicians		Nursing staff		Scientific research and management fellow	
	Urban	Rural	Urban	Rural	Urban	Rural
Academic degrees						
Doctor’s degree	566 (10%)	8 (1%)	233 (2%)	1 (1%)	159 (6%)	1 (1%)
Master’s degree	1 437 (25%)	46 (5%)			246 (10%)	4 (1%)
Bachelor’s degree	2 807 (50%)	398 (45%)	2 295 (23%)	173 (11%)	956 (38%)	147 (30%)
College degree	708 (13%)	335 (38%)	5 250 (52%)	726 (48%)	712 (28%)	211 (44%)
Technical secondary school education	130 (2%)	88 (10%)	2 228 (22%)	586 (39%)	258 (10%)	77 (16%)
Others	11 (1%)	12 (1%)	20 (1%)	9 (1%)	172 (7%)	41 (8%)
Professional ranks						
Senior level	1 915 (34%)	209 (24%)	237 (2%)	32 (2%)	248 (10%)	19 (4%)
Medium level	1 693 (30%)	278 (31%)	4 310 (43%)	512 (32%)	449 (18%)	95 (20%)
Junior level	2 051 (36%)	400 (45%)	5 251 (52%)	989 (61%)	539 (21%)	101 (21%)
Others	**/**	**/**	334 (3%)	79 (5%)	1 267 (51%)	266 (55%)

The average academic degree of physicians has been rising from technical secondary school education to bachelor’s degree, that of nurses has been rising from technical secondary school education to college degree, and that of scientific research and management personnel has been rising from college degree to bachelor’s degree. Regarding the scientific research and management fellows, their educational level was slightly higher among those working in urban institutions. In terms of professional level, physicians with senior, mid, and junior levels were equally distributed in urban institutions, while in rural ones, more physicians had a junior rank. Just 2% of nursing staff had senior professional ranks, and most of them were of junior or midlevel employees

### Regional distribution of ophthalmic human resources

Table 5 compares the number of health personnel in the eastern, central, and western parts of China. In the eastern region, physicians with medical degrees accounted for 13% of the total population, while regarding those working in the central and western regions, only 5% and 2% had medical degrees. As for scientific research and management fellows, only 8% of them, in the eastern regions, hold doctoral degrees; meanwhile, 1% and 2% of those in the central and western regions do in comparison.

**Table 5:** Human resource in ophthalmic medical institutions in eastern, central, and western China

	Physicians			Nursing staff			Scientific research and management fellow		
	Eastern	Central	Western	Eastern	Central	Western	Eastern	Central	Western
Academic degrees									
Doctor’s degree	444 (13%)	112 (5%)	18 (2%)	193 (3%)	41 (1%)	0 (0%)	145 (8%)	9 (1%)	6 (2%)
Master’s degree	970 (27%)	449 (21%)	64 (7%)				211 (11%)	27 (4%)	12 (4%)
Bachelor’s degree	1 574 (45%)	1 159 (54%)	472 (56%)	1 379 (23%)	948 (23%)	141 (9%)	692 (36%)	299 (40%)	112 (33%)
College degree	445 (12%)	354 (16%)	244 (28%)	2 987 (50%)	2 116 (53%)	873 (55%)	513 (27%)	262 (35%)	148 (44%)
Technical secondary school education	91 (2%)	77 (3%)	50 (6%)	1 345 (23%)	888 (22%)	581 (36%)	208 (11%)	93 (13%)	34 (10%)
Others	13 (1%)	8 (1%)	2 (1%)	24 (1%)	5 (1%)	0 (0%)	135 (7%)	54 (7%)	24 (7%)
Professional ranks									
Senior level	1 157 (33%)	725 (34%)	242 (29%)	132 (2%)	101 (2%)	36 (2%)	170 (9%)	65 (9%)	32 (10%)
Medium level	1 097 (31%)	642 (30%)	232 (27%)	2 842 (48%)	1 555 (38%)	425 (26%)	309 (16%)	152 (20%)	83 (25%)
Junior level	1 283 (36%)	792 (36%)	376 (44%)	2 766 (46%)	2 342 (57%)	1 132 (69%)	404 (21%)	175 (24%)	61 (18%)
Others	**/**	**/**	**/**	234 (4%)	120 (3%)	54 (3%)	1 021 (54%)	352 (47%)	160 (48%)

## Discussion

### Ophthalmic specialized hospitals develop rapidly supported by government at varied levels

On 3 December 2010, the State Development and Reform Commission, the former Ministry of Health, the Ministry of Finance, the Ministry of Commerce, and the Ministry of Human Resources and Social Security of People’s Republic of China jointly published the *Comments on Further Encouraging and Guiding Social Capital to Establish Medical Institutions*. It was named “the wind indicator for encouraging and supporting the development of non-public hospitals in China.” It advocated for the recognition of the significance that encouraging and guiding social capital to establish medical institutions can improve the medical practicing environment and promote sustainable and healthy development of non-public medical institutions [12].

Following its release, the Chinese national government issued many other policies in succession to encourage social capital to fund healthcare industry, creating a favorable environment for policies to be implemented. Based on *China’s Health Statistics Yearbook 2016*, the number of health personnel per thousand populations rose from 4.39 in 2010 to 5.56 in 2014. Additionally, the number of physicians and nursing staff per thousand populations rose from 1.80 to 2.12 and 1.53 to 2.20, respectively. The ratio of physicians to nurses in 2014 reached to 1:1.04, and the ratio of health personnel in public medical institutions to private ones was 4.53:1 [7]. Meanwhile, with the population of the elderly increasing in China, people’s needs for healthcare services are on the rise. Many also have concerns about the quality, accessibility, and management of medical services, such as hospital environment, patient visit process, and quality of medical care. As social funding pours into the market, specialized hospitals, especially non-public specialized hospitals, will flood into the market and fuel the period of rapid expansion. Based on *China Statistical Yearbook 2016*, data gathered from 2011 to 2015, the quantity of specialized hospitals rose from 4 283 to 6 023 [1].

In general hospitals, departments of ophthalmology often do not get enough funding or support, thereby slowing their development. On average, the department of ophthalmology accounted for just 2.5–5% of total revenue of general hospitals [7]. Specialized hospitals have an easier market entry experience, gain independence in management, and rapidly gain access to technology; as a result, many departments of ophthalmology become independent from general hospitals and set up eye centers or eye hospitals. Simultaneously, private and/or foreign capital has begun to flood into the medical industry in China and non-public hospitals have increased substantially [13, 14].

With the expansion of ophthalmic specialized hospitals, there has been a rise in the number and professional expertise of health personnel working in specialized hospitals. The market share of specialized ophthalmic hospitals continues to rise over the following years [14].

### Private ophthalmic medical institutions still face challenges in development, especially in terms of human resource allocation

Based on the 2015 survey, on average, each public ophthalmic medical institution has 41 physicians and 59 nursing staff, which is three times as much as those working in private institutions, with respectively 12 physicians and 24 nursing staff.

In addition to the difference in the relative number of health personnel, the percentage of personnel with higher academic degrees also differs. It is very clear that a majority of health personnel with the highest academic degrees work in public ophthalmic hospitals. In China, the history of public hospitals is long. Some are affiliated with large medical universities, so they often have better reputations. Meanwhile, the better working environment and higher per-capital income in urban areas and eastern region also draws a large group of talented health personnel. Moreover, those hospitals have a relatively mature talent training platform and rich teaching and research resources, attracting top-level medical talent. For the reasons listed above, many health personnel, especially young and highly educated ones, are scrambling to work in public hospitals [15, 16].

In terms of professional ranks, ophthalmic health personnel rank higher in public hospitals than in private institutions. The current professional title review system has been used in public hospitals as the main way for training and selecting talents since the 1980s. The process includes a personal statement, employer recommendations, review by a specialized department, and administrative department approval. Unified and objective indicators for review have been adopted in each hospital including post-tenure, foreign language training, computer software knowledge, quantity of published research papers, achievements in scientific research, and quantity and level of education reached [17]. As professional ranking is closely connected with salary, insurance, and other welfare incomes, ranking restricts the mobilization of health personnel to a large extent. Many people stay in one hospital or even one position for a lifetime.

However, since qualifications of private medical institutions differ, health personnel are often met with difficulties in academic titles conferring. Additionally, operating performance and economic benefit weigh higher than research achievement in private medical institutions, making it difficult for people working there to devote time to research work, which is needed for promotion in the professional ranks.

In private medical institutions, they often implement favorable policies to attract health personnel, but the shortage of talent is still the biggest obstacle to the comprehensive development. Aside from the difficulty they face in recruiting talent, they also have trouble retaining talent; staff turnover is very high. There are several reasons for that, including an undeveloped social security mechanism; a difficult to navigate promotion, recruitment, and retention system; an archaic economic benefit-oriented salary system, and a unified price setting system for medical service [18].

Few private hospitals are willing to cultivate and mentor health personnel because it requires a large time commitment and financial investment and has a high risk of burn out. Another concern is that there are no standard criteria for promotions, so fewer talented health personnel are willing to work in private hospitals. These hospitals, as a result, sometimes rely largely on hiring relatives and friends to expand their business. Therefore, health personnel in private hospitals are usually presented in a two-tier ranking system composed of high-ranked retired health personnel who act as the experts and recently graduated students who provide basic services, which forms unreasonable talent structure [15].

### The gap in human resources persists between urban and rural ophthalmic medical institutions

Based on the 2015 survey, we can find there is little difference in the average number of health personnel in rural vs. urban medical institutions. In every urban institution, there are 17 physicians and 30 nursing staff, compared to the 14 physicians and 26 nursing staff working in every rural institution. However, when examining the density of human resource allocation, it is evident that there is a large gap in the provision of health resources in certain regions. The number of physicians, nursing staff, and scientific research and management fellows per 10 000 population in 2014 was respectively 0.07, 0.13, and 0.03 in urban areas, while it was 0.02, 0.03, and 0.01 in rural areas.

The Chinese government pays close attention to and adopts many measures to promote the development of healthcare in rural areas. Since the new medical reform program in China was implemented in 2009, hierarchical medical and two-way referral systems have been vigorously promoted throughout the country. In this campaign, pairing-assistance mechanisms between urban and rural medical institutions have been highly effective. In 2014, the former China National Health and Family Planning Commission issued *Opinions for Further Deepening Pairing-Assistance between Urban and Rural Medical Institutions* and *Work Plan on Deepening Pairing-Assistance between Urban and Rural Medical Institutions from 2013 to 2015*. As a result, national projects were carried out accordingly, such as *Rural Health Assistance Campaign by Ten Thousands Physicians and Training Program for Essential Physicians in County Hospitals* [19, 20]. Comprehensive capacity building of county hospitals was also initiated. *Work Plan for Elevating Comprehensive Capacity in County Hospitals* was published in 2014, putting forward the goal of raising the outpatient care rate within counties to 90% [21]. Moreover, the criterion for medical service capability of county hospitals has been formulated and 500 county hospitals have been selected to pilot a comprehensive capacity building program. A variation of methods have been used to improve the service capability of medical institutions located in rural areas, including the wide use of tele-medicine and medical tours by national medical groups [22].

However, from this study, we can clearly see, although the absolute number of health personnel has increased, the relative number of human resources in specialized ophthalmic medical institutions is still quite low. They cannot meet the needs of the rural population of China. Reasons may include poor health system infrastructure, insufficient resources for further development, and unequitable treatment of talent in rural areas. Therefore, there is still a long way to go in order to achieve an equitable and regionally comprehensive health and medical development throughout China. More health resources should be allocated to rural areas to further improve medical service accessibility and the service capacity of rural medical institutions.

### Differences in human resource allocation are apparent regionally, which directly correlates with inequity and the inaccessibility of healthcare provision

Based on this study, the quantity and levels of expertise among ophthalmic health personnel differ by regions. The number of physicians, nursing staff, and scientific research and management fellows per 10 000 population in 2014 was respectively 0.06, 0.10, and 0.03 in the eastern region; 0.05, 0.10, and 0.02 in the central region; and 0.02, 0.04, and 0.01 in the western region. Regarding physicians with medical degrees, 13% practiced in eastern China, 5% in central China, and 2% in the west.

Since China has a vast territory with a large population, the phenomenon of unequal regional development has serious repercussions. Although the central government has adopted many strategies to develop the western and central regions of China, the regional differences are still apparent. In 2016, the per capita GDP of Tianjin (located in eastern region), which ranked the highest among all the 31 provinces, was about $18 257, which is four times the number of Gansu, a western province [1]. The differences in their economic development have led to a regional gap in health and medical resource allocation in the western providences.

In a paper published in 2014, four health and medical resource indicators were identified to evaluate resource allocation in 31 provinces of China, including the number of health workers, health institutions, medical beds, and financial subsidies per thousand population. The result showed there was a great difference in health resource allocation within provinces [23]. Similarly, another scientific paper published in 2016 reported a comprehensive evaluation of China’s healthcare system using 18 indicators in three categories: quantity of health institutions per 10 thousand population, healthcare personnel per 10 thousand population, and health expense per capital. The authors concluded that healthcare development in China was not regionally equitable and can be divided into five levels of capacity [24].

## Conclusion

In this study, we found that with the strong support of the Chinese government, specialized ophthalmic medical institutions have achieved great progress during the past 10 years. The number, educational level, and professional ranking of ophthalmic personnel rose greatly since the Chinese government implemented development programs. However, the difference between public and private, between urban and rural areas, and between eastern, central, and western regions is great. Inequity in the spatial and structural distribution of health services creates efficiency issues.

Therefore, in order to achieve health and health resource allocation equity nationally in China, its health talent recruitment structure needs to be improved. We suggest that more studies should be commissioned and adopted, to develop and to increase health human resources at the grassroots level. Additionally, the national policies and strategies that have been implemented need to be continually monitored and evaluated to test their effectiveness. The intended effect would be that the existing barriers in ophthalmic human resource allocation can be removed and national equity of health service would be achieved in China.

The large studies mentioned throughout this paper covered a broad number of topics, involving almost all ophthalmic medical institutions in China. Ophthalmic care professionals were divided into seven groups; for the purposes of this paper, we focused on physicians, nursing staff, and scientific research and management fellows to make a manageable comparative analysis. The three were the main groups of ophthalmic health personnel in China and accounted for the clear majority of personnel in ophthalmic facilities. Therefore, the analysis of those three groups can be representative of the overall ophthalmic human resource development in China.

As it is centered on China’s National Eye Health Strategy, this analysis is a significant tool that can be used to provide a scientific basis for the Chinese government to make decisions about health policy and achieve universal eye health. The study also has some limitations. Since it is a descriptive analysis, it may not address other potential factors which influence the work environment and quantity of health personnel in specialized ophthalmic medical institutions.

## Data Availability

The main dataset based on the Survey of China National Eye Care Capacity and Resource in 2015 is not publicly available due to requirement for data confidentiality and privacy in China but are available from the corresponding author on reasonable request. The data obtained from *China’s Health Statistics Yearbook 2016* is publicly available.
